# Non-histone lactylation in cancer: current advances and clinical implications

**DOI:** 10.3389/fimmu.2026.1824457

**Published:** 2026-05-28

**Authors:** Robert Le Yang, Yiran Liang

**Affiliations:** Department of Breast Surgery, General Surgery, Qilu Hospital of Shandong University, Jinan, Shandong, China

**Keywords:** non-histone, lactylation, post-translational modification, cancer, clinical implications

## Abstract

Lactylation, a novel post-translational modification characterized by the covalent attachment of lactate to specific lysine residues, has emerged as a critical metabolic regulator in cancer. Initially linked to histone modification-mediated transcriptional control, recent advancements have expanded this paradigm by revealing widespread non-histone lactylation, which modulates protein stability, enzymatic activity, and interactions, thereby significantly impacting tumor proliferation, metastasis, and therapy resistance. Recent advances have uncovered several key enzymes driving lactylation, including lysine acetyltransferases and deacetylases, and novel “writers” like alanyl-tRNA synthetases, highlighting the complexity and diversity of lactylation mechanisms. This review synthesizes current advances in non-histone lactylation in cancer, emphasizing its regulatory mechanisms and functional impacts. We also discuss potential therapeutic strategies, such as small-molecule inhibitors and peptides targeting lactate-driven pathways. By integrating current evidence, this review underscores non-histone lactylation as a promising area for cancer research and management, offering new insights into the complex interplay between metabolism and tumor biology.

## Introduction

Despite the fact that the human genome encodes approximately 20,000 genes, there are millions of proteins that are active within the human body. Post-translational modifications (PTMs), covalent alterations to amino acid residues that occur during or after protein biosynthesis, are significant contributors to the remarkable diversity of the proteome (1). Common PTMs, including methylation, acetylation, phosphorylation, and ubiquitination, play crucial roles in modulating protein stability, localization, enzymatic activity, and interaction networks, thereby influencing almost all biological processes, such as signal transduction, metabolism, and cell cycle control (2–4). For instance, phosphorylation serves as a key regulatory mechanism for enzyme activity and signaling pathways, while ubiquitination controls protein degradation via the ubiquitin-proteasome system (5). Dysregulated PTMs could contribute to oncogenic signaling, metabolic reprogramming, and therapeutic resistance, positioning them as central mediators in cancer.

A hallmark of cancer is the Warburg effect (aerobic glycolysis), wherein cancer cells preferentially utilize glycolysis for energy production even under oxygen-rich conditions. This glucose metabolic shift enhances the production of lactate, which accumulates in the tumor microenvironment (TME), facilitating immune evasion, angiogenesis, and metastatic spread (6). Beyond its role as a metabolic byproduct, lactate has recently been implicated in epigenetic and proteomic regulation via lactylation, a novel PTM involving the covalent attachment of lactate to lysine residues (Kla), which has recently gained attention in cancer research (7). Previous studies have consistently centered on histone lactylation, which modulates chromatin structure and transcriptional programs, thereby linking metabolic states to epigenetic regulation (8). For instance, histone H3K18 lactylation (H3K18la) in macrophages promotes pro-tumorigenic cytokine expression under hypoxic conditions (9). Moreover, H3K18la in cancer cells has been demonstrated to upregulate VCAM1 transcription and activate the AKT-mTOR signaling pathway, thereby promoting tumor cell proliferation and migration, which underscores the significance of the histone Kla/VCAM1/AKT-mTOR axis in metabolic regulation (10).

Recent breakthroughs have unveiled that lactylation extends far beyond histones, acting as a pivotal mechanism in cancer biology. A global lactylome profiling was obtained using LC-MS/MS, and 9,275 Kla sites were identified, of which 9,256 sites were located in non-histone proteins, suggesting that lactylation is a universal modification and expanding the understanding of its function (11). Non-histone targets, such as metabolic enzymes, signaling kinases, and molecular chaperones, undergo lactylation, which reprograms their functions to fuel tumor progression. For example, lactylation of pyruvate kinase M2 (PKM2) promotes its pyruvate kinase activity while preventing its nuclear translocation (12, 13). Emerging discoveries underscore the capacity of lactylation to reprogram protein functions, which serves as a critical bridge between tumor metabolism, transcriptional reprogramming, and cellular plasticity. Continued efforts to map site-level causality and develop actionable lactylation-directed interventions hold immense promise for overcoming therapy resistance and improving precision oncology (14, 15).

This review systematically synthesizes the advances in non-histone lactylation, focusing on its regulatory mechanisms, functional consequences, and clinical implications. We begin by exploring the metabolic origins of lactylation, dissect its crosstalk with oncogenic pathways, and highlight its role in driving metastasis and therapy resistance. Finally, we delineate emerging therapeutic strategies targeting lactylation, from small-molecule inhibitors of enzymes to peptides blocking lactylation-dependent pathways, and outline future directions for translating these insights into clinical practice. Collectively, this review highlights non-histone lactylation as a linchpin connecting metabolic dysregulation and oncogenic plasticity, while proposing novel orientation for clinical interventions in aggressive tumors.

## Lactate metabolism and the catalytic mechanisms of protein lactylation

### Overview of lactate as a modification substrate

In cancer cells, glucose is preferentially converted to lactate even under normoxic conditions (the Warburg effect), a metabolic reprogramming that supports rapid proliferation and biosynthesis (16, 17). This process converts glucose to pyruvate, generating ATP, and pyruvate is then reduced to lactate by lactate dehydrogenase A (LDHA) (18). This high glycolytic flux drives lactate production at rates 10 to 100 times faster than the rate of complete mitochondrial glucose oxidation. To prevent intracellular acidification, the accumulated lactate is actively exported into the tumor microenvironment (TME) via monocarboxylate transporters, primarily MCT4. Conversely, MCT1 facilitates lactate uptake in adjacent oxidative tumor cells (19, 20). Although lactate can be taken up by neighboring cells, converted back to pyruvate (by LDHB), and subsequently enter the tricarboxylic acid (TCA) cycle after conversion to acetyl−CoA, its primary fate in most tumor cells is secretion, creating a lactate−rich niche (21, 22). Moreover, the intracellular pyruvate/lactate ratio mirrors the cellular redox status (NAD+/NADH balance), acting as a metabolic rheostat for adapting to oxidative stress or nutrient deprivation (23).

**Figure 1:**
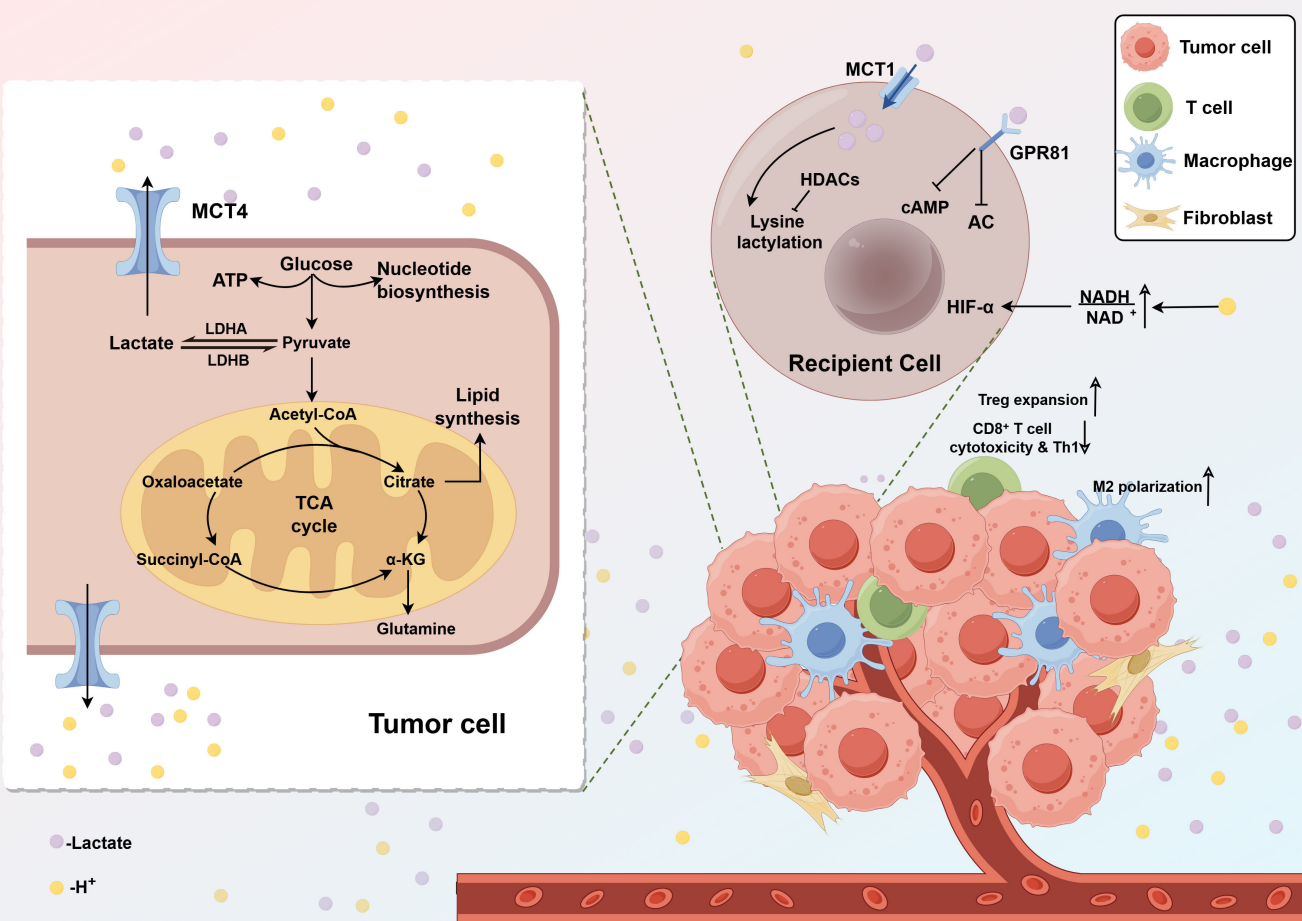
Metabolic and signaling interplay of lactate between tumor cells and the tumor microenvironment. Under aerobic glycolysis, tumor cells convert pyruvate to lactate mainly through lactate dehydrogenase A (LDHA). Lactate is exported from highly glycolytic tumor cells predominantly through monocarboxylate transporters, especially MCT4. In the tumor microenvironment, extracellular lactate can be imported by neighboring tumor, stromal, endothelial, or immune cells through MCT family transporters (e.g., MCT1), or function as a signaling metabolite by activating the lactate receptor GPR81. After cellular uptake, lactate can be oxidized back to pyruvate via LDHB and enter mitochondrial metabolism through pyruvate-derived acetyl-CoA, thereby contributing to the TCA cycle and metabolic remodeling. In parallel, intracellular lactate serves as the direct biochemical substrate for lysine lactylation, which links tumor metabolism to epigenetic regulation and cell-state changes. Furthermore, extracellular lactate accumulation drives an immunosuppressive niche by impairing CD8+ T cell cytotoxicity and CD4^+^ Th1 polarization, promoting Treg expansion, and inducing M2 macrophage polarization. Lactylation dynamics are modulated by specific enzymes, with HDACs functioning as delactylases to erase lactylation marks. Lactate thus acts not only as a metabolic fuel but also as a signaling and epigenetic regulator that links tumor metabolism with microenvironmental remodeling. Cell types are labeled in the figure (red: tumor cells; green: T cells; blue: macrophages; beige: fibroblasts).

Extracellular lactate is now widely recognized as a signaling molecule, notably activating GPR81 (a specific lactate-sensing G protein−coupled receptor) and inducing acidosis within the TME (24). This acidic environment exerts broad immunosuppressive effects. For example, lactate impairs CD8^+^ T cell cytotoxicity by inhibiting TCR signaling and IFN−γ production (25, 26). It also skews CD4^+^ T cell polarization away from the antitumor Th1 phenotype via SIRT1−mediated deacetylation and degradation of T−bet (27). Additionally, lactate promotes the expansion and suppressive function of regulatory T cells (Tregs) (28, 29). In macrophages, lactate drives polarization toward a pro−tumor M2 phenotype (30) while suppressing anti-tumor M1 functions (16). Dendritic cell antigen presentation is compromised by lactate−induced downregulation of MHC−II expression and type I IFN production (31, 32). Furthermore, lactate suppresses NK cell cytolytic activity by blocking NFAT activation or disrupting lipid biosynthesis (33, 34). Collectively, these mechanisms establish an immunosuppressive niche that facilitates tumor progression (**Figure 1**).

However, recent breakthroughs have expanded our understanding of lactate far beyond its role as a metabolic byproduct or a microenvironmental signaling molecule. Crucially, intracellular lactate serves as the direct biochemical substrate for a novel post-translational modification (PTM), lysine lactylation (Kla) (35). This novel PTM directly links metabolic rewiring to proteomic and epigenetic regulation, establishing lactylation as a critical and emerging regulator of cancer biology.

### Catalytic mechanisms of lactylation

**Figure 2:**
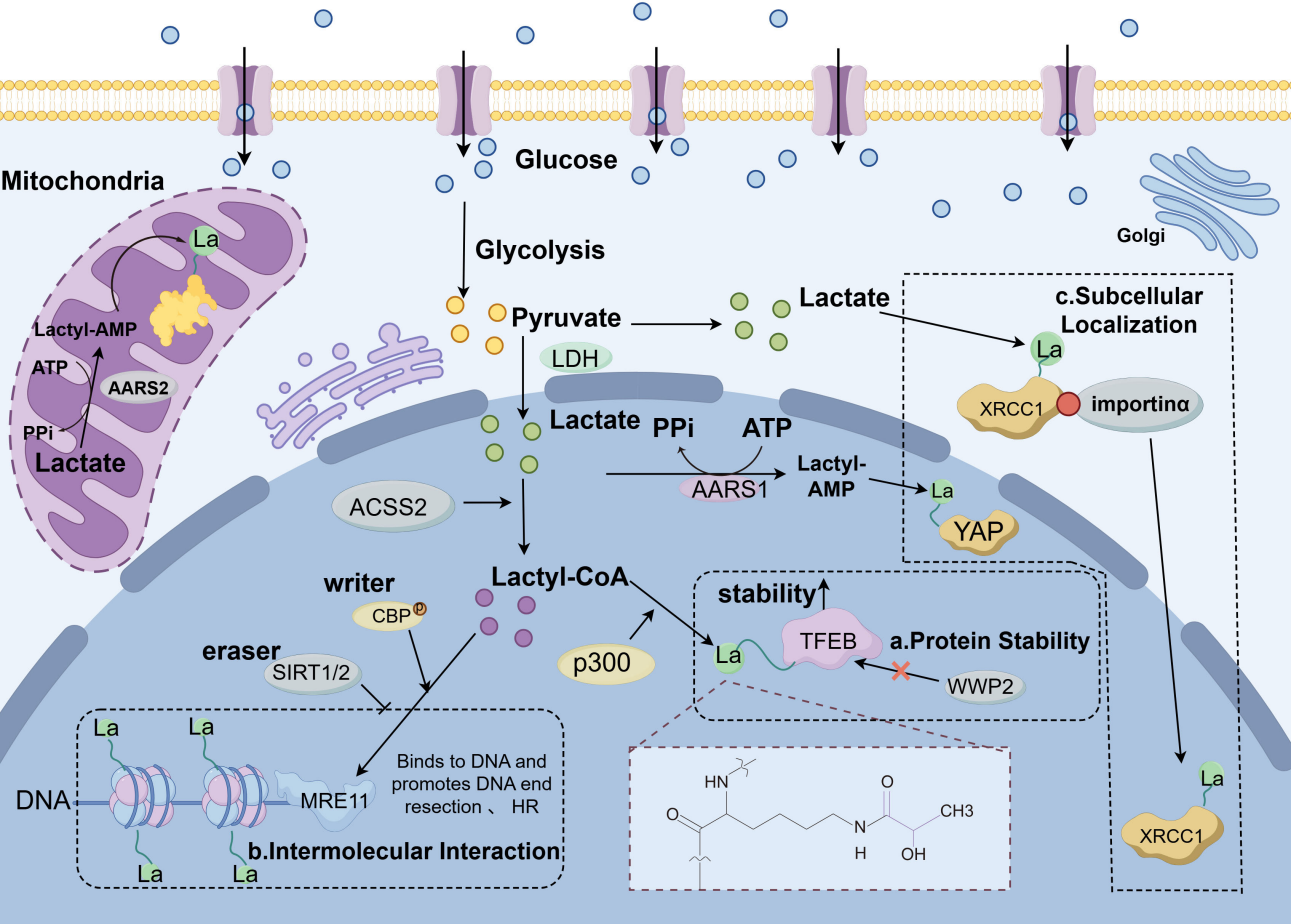
Regulatory mechanisms and functional consequences of protein lactylation. Glucose-derived pyruvate is converted to lactate by lactate dehydrogenase (LDH), and lactate serves as the substrate for protein lactylation through CoA−dependent and CoA−independent pathways. ACSS2 converts lactate into lactyl-CoA, which is used by writers such as p300/CBP to add lactoyl groups to lysine residues. AARS1 mediates CoA-independent lactylation by producing lactyl-AMP from lactate and ATP, and mitochondrial AARS2 may participate in lactylation within mitochondria. Erasers such as SIRT1/2 function as delactylases to remove lactyl groups. Lactylation regulates protein function by modulating multiple protein properties: **(a)** Protein Stability, lactylation stabilizes TFEB and counteracts WWP2-associated degradation; **(b)** Intermolecular Interaction, lactylation promotes MRE11 binding to DNA; and **(c)** Subcellular Localization, lactylation enhances XRCC1 nuclear localization via importin α. This diagram summarizes the enzymatic and non−enzymatic pathways of lysine lactylation (Kla) and its downstream effects on protein properties. This integrated view highlights how lactylation dynamically rewires protein function.

At the molecular level, lactylation is characterized by the covalent linkage between the carboxyl group (COOH) of lactate and the amino group (NH2) of a lysine residue (36, 37). This modification is primarily driven by two delineated mechanisms: enzymatic and non-enzymatic lactylation. Both pathways utilize lactic acid as a substrate but differ in chiral specificity: L-lactic acid participates in enzymatic lactylation, while D-lactic acid is linked to non-enzymatic processes. In addition, the regulatory mechanism is further complicated by the interplay of metabolic pathways, enzyme activities, and epigenetic modifications. The following section aims to synthesize the key regulatory pathways and related enzymes involved in lactylation, highlighting their roles in cancer biology (**Figure 2**).

#### Enzymatic lactylation

The majority of lactylation modifications identified thus far are enzymatic, which can be divided into CoA-dependent and CoA-independent mechanisms.

#### CoA-dependent pathways

Inspired by the similarities between lactylation and acetylation, researchers first identified the lactylation pathway via L-lactyl-CoA. Enzymatic lactylation primarily utilizes L-lactic acid as a substrate. Lactic acid is first converted to L-lactyl-CoA, which then serves as a donor to transfer lactoyl groups (CH3-CHOH-CO-) to lysine residues on specific substrates, including histones and other non-histone proteins (38). This process is catalyzed by a group of enzymes known as lactyltransferases, including p300, MOF, ME-PCT, and RE-PCT (9, 39, 40). While these ‘writers’ add the lactoyl groups, the dynamic reversal of this modification is governed by specific ‘erasers’, which will be discussed in detail below.

Previous studies have shown that lactylation and acetylation share similar processes of occurrence (8); thus, lactylation can use the same writers and erasers as acetylation. p300, the first identified lactylation writer, exemplifies functional versatility as an epigenetic writer, as it is shared by both acetylation and lactylation pathways via transferring acetyl or lactoyl groups to lysine residues, respectively (41). This overlap raises the possibility that other enzymes with established roles in writing, erasing, or reading classical epigenetic marks may similarly regulate lactylation. Guanosine triphosphate (GTP)-specific succinyl-CoA synthetase (GTPSCS) has been identified as a lactoyl-CoA synthetase in the nucleus, where it interacts with p300 to catalyze histone lactylation. The interaction with p300 relies on the nuclear localization signal of the G1 subunit and acetylation of K73 residue in the G2 subunit of GTPSCS, which synergistically induces histone H3K18la and GDF15 expression, promoting proliferation and radiation resistance in gliomas (42). In addition to acetyltransferases, a number of lactylation-specific enzymes have been identified. For instance, ACSS2 functions as a bona fide lactyl-CoA synthetase, facilitating the conversion of lactate to lactyl-CoA, which bridges lactate metabolism to KAT2-mediated lactylation (43).

#### CoA-independent pathways

Recent breakthroughs reveal a paradigm-shifting mechanism in which AARS1/2 (alanyl-tRNA synthetases) function as lactyl transferases, directly catalyzing ATP-dependent lactylation of L-lactate at lysine residues without requiring lactyl-CoA. Given the structural similarity between lactic acid and L-alanine, Ju et al. postulated the reaction process: the ATP-dependent lactate-AMP formation facilitates the transfer of lactate to covalently bound lysine residues, thereby mediating protein lysine lactylation (44). This report constitutes the initial documentation of a CoA-independent catalytic reaction process since the initial discovery of lactylation. AARS1 serves as an intracellular lactate sensor, capable of directly binding lactate and further catalyzing p53 lactylation (45). Additionally, AARS1 could also catalyze lactylation of key components of the Hippo signaling pathway (i.e., YAP and TEAD), which promotes their nuclear localization and further enhances the transcriptional expression of downstream genes and malignant proliferation in gastric cancer (46). AARS2, primarily located in mitochondria, complements the cytoplasmic function of AARS1 to facilitate mitochondrial protein lactylation, linking lactate metabolism to organelle function (47).

The efficiency of lactylation by acetyltransferases (e.g. p300) might be significantly limited by the fact that lactyl-CoA is barely detectable in tumor cells, with concentrations only 1/1000 that of intracellular acetyl-CoA (38). Therefore, the CoA-independent pathway is critical in tumors, positioning AARS1/2 as dominant lactylation writers in cancer contexts.

#### Non-enzymatic lactylation

Non-enzymatic lactylation involves the presence of D-lactic acid and does not require enzyme catalysis, often occurring spontaneously under certain metabolic conditions (48). D-lactic acid is often derived from glycolytic byproducts like Methylglyoxal (MGO). MGO forms lactyl-glutathione, which serves as a reactive intermediate enabling direct lysine modification (49). This process is amplified in the acidic tumor microenvironment (pH 6.5-6.9), where lactate accumulation drives spontaneous lactylation (50). Unlike enzymatic pathways, non-enzymatic modifications lack strict substrate specificity, potentially modifying diverse lysine residues across the proteome. Therefore, non-enzymatic lactylation amplifies global protein lactylation in cancer cells, synergizing with enzymatic pathways to reshape cellular signaling and PTM landscape.

### Erasers of lactylation

The removal of lactoyl groups, termed delactylation, is a crucial process that ensures lactylation remains a transient and responsive signal rather than a permanent state. This enzymatic reversal is primarily mediated by two families of deacetylases: the NAD^+^-dependent sirtuins (SIRT1-3) and the zinc-dependent class I histone deacetylases (HDAC1-3) (39, 51, 52), which are collectively termed “erasers”.

Recent studies have highlighted the distinct substrate specificities of Sirtuins toward lysine lactylation (Kla). For instance, proteomic analysis in SIRT1/SIRT3 knockout HepG2 cells revealed that while ENO1-K228la is a common target of both SIRT1 and SIRT3, PKM2-K207la is uniquely regulated by SIRT1 (53). Mechanistically, SIRT1-mediated delactylation of PKM2 restores its enzymatic activity, thereby enhancing glycolysis and cell proliferation. Beyond metabolic enzymes, SIRT2 has been identified as a delactylase for the m^6^A methyltransferase METTL16, which inhibits cuproptosis and subsequently reduces the therapeutic efficacy of the copper ionophore elesclomol (54). Furthermore, SIRT3-dependent delactylation of cyclin E2 modulates cell cycle progression, thereby preventing the growth of hepatocellular carcinoma (55).

Regarding HDACs, emerging studies have uncovered their specific non-histone substrates. HDAC3 has been identified as an eraser for NBS1-K388la, thereby disrupting MRN complex formation and dampening homologous recombination repair (56). Conversely, HDAC2−mediated delactylation of METTL3 enhances its interaction with WTAP, promoting m^6^A modification of DNA damage repair transcripts and driving chemoresistance in triple−negative breast cancer (57). Furthermore, in bladder cancer, HDAC2 acts as a global regulator of the lactylome, particularly by delactylating DHX15 to modulate RNA splicing and promote malignancy (58). Together, these erasers act as critical metabolic checkpoints, fine-tuning the cellular response to lactate-derived epigenetic and post-translational modifications.

### Readers of lactylation

The functional outcomes of lactylation depend critically on specialized “readers” that recognize lactylated lysines, translating chemical marks into precise biological consequences in a highly context-dependent manner (59–61). Currently, research on lactylation readers has predominantly focused on histones, given that histone modifications can contribute to the gene expression programs by affecting histone-DNA interactions or by recruiting specific protein effectors (readers) (62–64). Therefore, the interpretation of effectors reading histone lactylation is key to understanding the mechanism underlying epigenetic regulatory pathways.

The bromodomain (BRD) family, initially identified as a reader for histone acetylation, also exhibits affinity for lactylated histones (65, 66). BRD4, an essential member of the BRD family, possesses two bromodomains capable of recognizing lysine residues (66). The recognition of histone (e.g. H4K8la) by BRD4 enables the crosstalk between glycolysis and transcriptional regulation (67). Additionally, recent studies have shown that DPF2 can also recognize H3K14la and co-localize with it at oncogene promoters (e.g., MYC), recruiting chromatin remodelers to regulate specific gene transcription and influence biological outcomes (68). However, due to the conserved chemical nature of lactylated lysine residues across substrates, where the lactoyl group (CH3-CHOH-CO-) forms analogous interactions with reader domains, it is plausible that the readers for histone lactylation may also recognize and interact with non-histone lactylated proteins. These findings raise the possible existence of pan-lactylation readers, capable of decoding lactylation marks on both histones and non-histone proteins. Such shared readers could enable coordinated regulation of nuclear and cytoplasmic processes, linking lactate-driven epigenetic reprogramming to broader cellular signaling networks. Future research could further prioritize the identification and characterization of readers for non-histone lactylation to unravel their role in the cancer context.

## Molecular effects of non-histone lactylation and PTM crosstalk

Having established how lactate is generated and how the lactylation mark is written, read, and erased, we now examine the molecular consequences of lactylation on protein function. Beyond the simple addition of a lactoyl group, this modification can profoundly alter protein properties, including stability, enzymatic activity, intermolecular interactions, and subcellular localization, and can engage in intricate crosstalk with other post−translational modifications (PTMs) such as acetylation, methylation, phosphorylation, and ubiquitination (**Figure 2**). These molecular events form the mechanistic basis for the pleiotropic roles of non−histone lactylation in cancer.

### Alterations in protein characteristics

#### Protein stability

Lactylation exerts bidirectional control over protein stability, either enhancing or destabilizing targets depending on cellular context and modification sites.

A series of additional global lactate proteomic and metabolomic analyses were performed on samples from patients with non-small cell lung cancer (NSCLC). Through multi-omics analysis and *in vitro* and *in vivo* validation, intracellular lactate was found to induce lactylation of APOC2 at K70, which enhanced its stability and promoted extracellular lipolysis to generate free fatty acids, facilitating tumor metastasis and immunotherapy resistance (69). Additionally, MCT1-mediated extracellular lactate influx promoted the lactylation of HIF-1α, further stabilizing HIF-1α under normoxia in prostate cancer (70). Using LC-MS/MS, sequence alignments, and molecular docking, K172 was identified as the major lactylation modification site in DCBLD1 (71). Moreover, the mutant DCBLD1 (K172A) significantly inhibited its lactylation and further shortened its half-life, indicating that lactylation can directly stabilize DCBLD1 expression in cervical cancer. Lactylation of TFEB at K91 (TFEB K91la) enhances its protein stability by blocking WWP2-mediated ubiquitination and proteasomal degradation (72).

Another study amplifies the regulatory role of lactylation in protein stability. Lactylation of cyclic GMP-AMP synthase (cGAS) at lysine 21 (K21) directly destabilizes the protein by triggering its proteasomal degradation through non-canonical degradation pathways. Specifically, K21 lactylation redirects cGAS from the nucleus to the proteasome for degradation, which bypasses ubiquitination. Concurrently, PIK3CB lactylation at K415 rewires its activity, which impairs ULK1-mediated phosphorylation of proteasomal subunit PSMA4 (S188), further amplifying cGAS destabilization (73). These dual modifications orchestrate a “lactylation code” that synergistically governs cGAS stability, silencing cytosolic DNA sensing and suppressing interferon (IFN) production, thereby fostering immune evasion and tumor progression.

#### Enzymatic activity

The lactylation of certain enzymes has been demonstrated to influence their own activity and the related modulation process. A notable illustration of this dynamic interplay is the case of METTL family proteins, which function as methylation transferase enzymes. These proteins are susceptible to lactylation modification, subsequently affecting METTL enzyme activity and methylation levels of downstream targets. METTL16, an atypical methyltransferase, plays a pivotal role in the process of RNA N6-methyladenosine (m6A) modification (74). Recent research revealed that copper stress led to elevated lactylation of METTL16 at K229, which did not affect the nuclear localization of METTL16 but disrupted its autoinhibited state by altering the conformation of the E226 side chain, thereby enhancing its enzymatic activity and the m6A modification on FDX1 mRNA (54). Significantly, the augmentation of METTL16 lactylation can improve the therapeutic efficacy of the copper ionophore elesclomol, providing a promising combination treatment strategy for gastric cancer (54). As the final rate-limiting enzyme of glycolysis, PKM2 is responsible for converting phosphoenolpyruvate (PEP) to pyruvate while generating ATP (75). Unlike other isoforms, PKM2 exists in dynamic tetrameric and dimeric states, allowing metabolic flexibility to support rapid cell proliferation (76). Previous research has indicated that lactylation of PKM2 at K62 increases its pyruvate kinase activity by inhibiting the tetramer-to-dimer transition, further modulating glycolytic flux and ATP production (12).

In addition to the promoting effect, the inhibitory effect of lactylation on enzyme activity is also a conceivable possibility. A recent study by Gao et al. explored the role of mitochondrial pyruvate carrier 1 (MPC1) in regulating the lactylation of fatty acid synthase (FASN) and its impact on non-alcoholic fatty liver disease (NAFLD). The results of this study suggest that MPC1-mediated lactylation of FASN at K673 leads to a reduction in FASN activity, exacerbating lipid accumulation in NAFLD (77). Another study reported that hypoxia can induce the lactylation of mitochondrial proteins to constrain oxidative phosphorylation (OXPHOS) in muscle cells. Mechanistically, hypoxia promotes AARS2 accumulation to lactylate PDHA1 at K336 and carnitine palmitoyltransferase 2 (CPT2) at K457/8, inactivating both enzymes and inhibiting OXPHOS by limiting the inflow of acetyl-CoA from pyruvate and fatty acid oxidation, respectively. This modification helps balance reduced oxygen supply with oxidative phosphorylation activity, allowing cells to adapt to fluctuating oxygen levels and reduces energy expenditure during high-intensity endurance activity (47).

The above studies highlight the bidirectional regulatory effect of lactylation on the enzyme activity of targeted proteins, depending on substrate and microenvironmental cues.

#### Intermolecular interaction

Lactylation modulates macromolecular interactions by altering protein-protein and protein-nucleic acid binding affinities, with profound implications for the modulation of cellular processes including mitochondrial dynamics, signal transduction, epitranscriptomic regulation, genomic stability, and treatment resistance.

Mitochondria are highly dynamic organelles that maintain cellular homeostasis through a balance of fusion and fission processes (78). Disturbance in the balance, particularly excessive mitochondrial fission, can alter energy metabolism, redox balance, and apoptosis, contributing to various diseases. Recent research has highlighted the role of lactylation in regulating mitochondrial dynamics through its effects on the Mitochondrial Fission 1 (Fis1), which is a key protein involved in mitochondrial fission (79). Increased levels of intracellular lactate can promote lactylation of Fis1 at lysine 20 (Fis1 K20la) in renal tubular epithelial cells, which strengthens its interaction with dynamin-related protein 1 (DRP1), triggering excessive mitochondrial fission. The resulting mitochondrial fragmentation leads to ATP depletion, mitochondrial reactive oxygen species (mtROS) overproduction, and activation of mitochondrial apoptosis pathways, which collectively exacerbate sepsis-associated acute kidney injury (79).

MOESIN (membrane-organizing extension spike protein) plays significant roles in a variety of diseases through bridging membrane dynamics to cellular behaviors, such as cell shape regulation, signal transduction, and cell trafficking (80, 81). Notably, the lactylation of MOESIN at K72 leads to enhanced binding to transforming growth factor β (TGF-β) receptor I. This interaction further facilitates downstream SMAD3 signaling, driving epithelial-mesenchymal transition (EMT) and tumor progression in cancers (82).

The impact of lactylation extends to RNA metabolism and DNA repair. METTL3, another central m6A methyltransferase, plays a crucial role in gene expression regulation and immune response (83). Recent research demonstrated that lactylation of METTL3 significantly impacts its function, particularly in the context of tumor-infiltrating myeloid cells (84). Using a colorimetric method to determine the enzymatic activity, the study found that the lactylation at K281/345 on METTL3 did not influence its enzymatic activity. Notably, METTL3 lactylation was shown to alter its conformation, thereby enhancing its interaction with RNA substrates, which further amplifies the m6A methylation levels and translation efficiency of downstream targets like JAK1 (84). In the context of genomic stability, lactylation of the homologous recombination (HR) repair protein MRE11 at lysine 678 (K678la) by CBP acetyltransferase enhances its DNA-binding capacity. This modification accelerates DNA end resection, a critical step in HR repair, thereby maintaining genomic integrity under DNA damage conditions (85).

In pancreatic ductal adenocarcinoma (PDAC), increased global protein lactylation correlates with poorer response to immunotherapy based on the analysis of ^18^F-FDG uptake (86). Specifically, lactate induces lactylation of endosulfine alpha (ENSA) at K63, which enhances its interaction with the PP2A regulatory subunit B delta isoform (PPP2R2D), thus inhibiting the catalytic activity of PP2A and triggering STAT3-CCL2 signaling (87). Therefore, the interaction between ENSA and PPP2R2D promotes the CCL2 secretion and tumor-associated macrophage (TAM) recruitment, inducing an immunosuppressive environment.

#### Subcellular localization

Lactylation dynamically regulates protein trafficking and compartmentalization, linking metabolic states to spatial control of signaling molecules. For instance, lactylation of PKM2 at lysine 62 stabilizes its tetrameric conformation but reduces its dimer formation. This shift promotes its pyruvate kinase activity, while attenuating its nuclear distribution and regulatory effects on the expression of pro-glycolytic enzymes, contributing to the modulation of inflammatory metabolic adaptation in pro-inflammatory macrophages (12, 88). Another study by Du et al. examined the function of hepatocyte HSPA12A in liver ischemia/reperfusion (I/R) injury (89). The study reveals that lactate induces lactylation of high-mobility group box 1 (HMGB1) to promote its cytoplasmic accumulation and subsequent sequestration into lysosomes. This lysosomal sorting facilitates HMGB1 release via exosomes, which further dampens macrophage chemotaxis and activation, thereby alleviating inflammatory damage during liver I/R (89).

Conversely, MCT1-mediated lactate influx drives lactylation of the transcription factor Snail, triggering its nuclear localization (90). Nuclear Snail amplifies TGF-β signaling by enhancing SMAD3/4 complex formation, which accelerates EMT and metastatic dissemination (90). In glioblastoma, XRCC1 undergoes lactylation at K247, a modification that significantly strengthens its binding affinity for Importin α, thereby driving its nuclear translocation and facilitating DNA repair (91). These examples illustrate how lactylation serves as a metabolic rheostat, spatially redistributing proteins to regulate anabolic demands, stress adaptation, and metastasis.

### Crosstalk with other post-translational modifications

The interplay between lactylation and other PTMs (e.g., acetylation, methylation, phosphorylation, ubiquitination) creates a dynamic and complex regulatory network that integrates metabolic cues with signaling outputs. Driven by shared substrates, enzymes, spatial-temporal competition, and regulation on downstream targets, this crosstalk establishes non-histone lactylation as a metabolic “rheostat” that fine-tunes broad PTM landscapes in response to cellular energy states, with profound implications for tumor progression (47).

#### Acetylation

The interplay between lactylation and acetylation, two lysine-targeting PTMs, manifests as context-dependent competition and functional collaboration (92). The ratio of lactylation to acetylation serves as a dynamic biosensor in cellular metabolism, mirroring intracellular lactate and acetyl-CoA concentrations while concurrently indicating the predominant metabolic mode.

Because lactoyl and acetyl groups share similar structural properties, they directly compete for the same lysine residues (9). While histone lactylation has been extensively studied (93), emerging evidence illustrates that this direct competition also occurs on non-histone substrates. A prominent example is the tumor suppressor p53, where K120 lactylation (K120la) competitively suppresses K120 acetylation (K120ac), thereby blocking its transcriptional activation of apoptosis-related genes (45). Paradoxically, these PTMs also collaborate to modify the same or different proteins through shared enzymatic “writers” (e.g., p300) and partial overlap in “erasers” (e.g., HDAC1/3) (39, 52). For example, HMGB1 undergoes both lactylation and acetylation under polymicrobial sepsis, and the two modifications synergistically amplify alarmin signaling, linking metabolic stress to systemic inflammation (89).

The balance between competition and synergy is metabolically gated and context-dependent. In highly glycolytic conditions, elevated lactate drives lactylation, whereas acetyl-CoA abundance favors acetylation. This dynamic duality extends across non-histone substrates, where lactylation may override acetylation-mediated activation or repression, creating a metabolic “switch” for tumor plasticity.

#### Methylation

While direct competition between lactylation and methylation on the same lysine residues has been classically characterized in histones (94), crosstalk between lactylation and methylation on non−histone proteins remains largely elusive. Instead, emerging evidence reveals that non-histone lactylation acts as an upstream trigger to drive protein methylation cascades and modulate RNA methylation machinery.

For instance, under hypoxic conditions, protein arginine methyltransferase 1 (PRMT1) undergoes lactylation at conserved residues K134 and K145. This modification enhances its methyltransferase activity, subsequently catalyzing the asymmetric dimethylation (aDMA) of the cytoskeletal protein vimentin at arginine 64 (R64). By promoting vimentin filament assembly, this lactylation-protein methylation axis directly drives cytoskeletal remodeling and tumor metastasis in triple-negative breast cancer (95). In the context of epitranscriptomic regulation, lactylation of the m6A writer RBM15 at K850 inhibits its proteasome-mediated degradation and enhances its association with METTL3, leading to increased global m6A methylation levels and promoting lung adenocarcinoma progression (96). Similarly, in colorectal cancer, K92 lactylation of the RNA-binding protein YBX1, installed by p300 and erased by SIRT2, significantly enhances its ability to recognize and associate with m5C-modified transcripts, a function critical for YBX1−driven malignant behaviors (97).

Together, these examples illustrate how non-histone lactylation serves as a master regulator bridging metabolic shifts to both proteomic and transcriptomic methylation networks. However, direct competition between lactylation and methylation on shared lysine residues of non−histone proteins remains largely unexplored and warrants future investigation.

#### Phosphorylation

The crosstalk between phosphorylation and lactylation constitutes a metabolic-signaling nexus that dynamically regulates cellular fate, with functional consequences spanning oncogenesis, DNA repair, and metabolic homeostasis. Significantly, phosphorylation frequently primes Kla modifications by activating key metabolic enzymes. For instance, phosphorylation of LDHA at Y10 by FGFR1 kinase stimulates its catalytic activity, amplifying lactate production to fuel non-histone Kla (e.g., VPS34 K781la, which inhibits autophagosome maturation) (98, 99). Additionally, CBP-mediated lactylation of MRE11 is dependent on ATM kinase phosphorylation, enhancing DNA repair ability (85). Nevertheless, the relationship between phosphorylation and lactylation is intricate, characterized by both cooperative and antagonistic impacts on the modifications of target proteins. Lactylation of transcription factor Sox10 is contingent on its prior phosphorylation, modulating the transcriptional program of VSMC transdifferentiation and pyroptosis (100). Conversely, AMPKα Kla suppresses its phosphorylation, inducing senescence while inhibiting autophagy under glutamine deprivation (101).

The duality of the interplay between phosphorylation and lactylation might be governed by the structural determinants of specific substrates, manifesting as cellular context-dependent phenotypic outputs. Deciphering the mechanisms underlying their crosstalk is essential for unraveling the integration of cellular metabolic and signaling pathways.

#### Ubiquitination and ubiquitin-like modification

Current studies reveal that lactylation and ubiquitination are involved in multi-dimensional interactions to regulate protein stability, mainly through three distinct tiers (41, 102).

Lactylation participates in the regulation of the expression of ubiquitin enzymes. For example, the lactylation of USP14 at lysine 336 is elevated in HCC, indicating crosstalk between these two modifications (103).

Additionally, there exists direct lactylation-mediated functional interference on ubiquitination. Meng et al. found that lactate treatment led to increased global lactylation while decreasing global ubiquitination. Lactylation of DCBLD1 at K172 blocks ubiquitin-mediated degradation, prolonging its stability and pro-metastatic activity in cervical cancer (71). Similarly, HIF-1α lactylation at specific lysine residues competes with ubiquitination, stabilizing HIF-1α protein by preventing its degradation and enhancing the expression of downstream genes involved in angiogenesis and glycolysis (70, 104). Further extending this paradigm, NSUN2 lactylation at K692 blocks its ubiquitination and proteasomal degradation, stabilizing the protein and promoting perineural invasion in pancreatic cancer (105). In colorectal cancer, NOL6 undergoes lactylation at K54, which inhibits its ubiquitination and degradation. Stabilized NOL6 then recruits deubiquitinase STAMBP to remove K48-linked polyubiquitin chains from transcription factor YY1 at lysine 339, preventing YY1 degradation and enhancing c-Myc transcription. A feed−forward loop is formed where c-Myc binds to the NOL6 promoter, reinforcing NOL6 expression and driving CRC proliferation and metastasis (106). Conversely, hypoxia-induced lactylation promotes Axin1 ubiquitination, subsequently enhancing glycolysis and stemness in esophageal cancer (107).

Lactylation can also cause allosteric disruption of E3 ligase-substrate interactions. K33la hinders the binding capacity of NEDD4 with caspase-11, inhibiting ubiquitination of caspase-11 and enhancing pyroptosis (108). Furthermore, lactylation of TFEB at K91 disrupts its interaction with E3 ubiquitin ligase WWP2, thereby inhibiting TFEB ubiquitination and subsequently elevating autophagic flux (72).

#### Other acylated modifications

Emerging evidence reveals that lactylation operates within a multi-acyl modification network, co-occurring with diverse acylations on the same protein, such as crotonylation, malonylation, and succinylation, thereby co-regulating the expression and function of the target proteins.

Proteome-wide analyses across species demonstrate this phenomenon. For instance, in rice, 60% of lactylated proteins are concurrently 2-hydroxyisobutylated, 33% succinylated, 29% acetylated, and 25% malonylated, with 49 proteins co-modified by five acylation modifications, indicating that many lysine residues can be modified by multiple acyl groups (109). In Botrytis cinerea, 143 lysine sites on 83 lactylated proteins are co-modified by crotonylation and 2-hydroxyisobutyrylation, further suggesting the evolutionary conservation of acyl crosstalk from fungi to plants and humans (110). Additionally, the shared enzymatic writers like p300, capable of depositing multiple acyl groups, act as PTM integrators to cooperatively regulate protein functions (111). However, the determinants of acyl group selection remain enigmatic, which might be governed by metabolite abundances and structural plasticity.

Collectively, lactate transcends its role as a mere metabolic byproduct, emerging as a significant regulator of complex PTM cross-communication. However, while most of the currently reported relationships between different PTMs are concerned with upstream and downstream pathways, direct competition at shared lysine sites remains underexplored, representing a critical knowledge gap. Future research should focus on further elucidating the cellular context-dependent PTM hierarchies and the functional consequences, as well as exploring the potential therapeutic targets within this interplay to enhance therapeutic efficacy.

## Non-histone protein lactylation and cancer hallmarks

**Figure 3:**
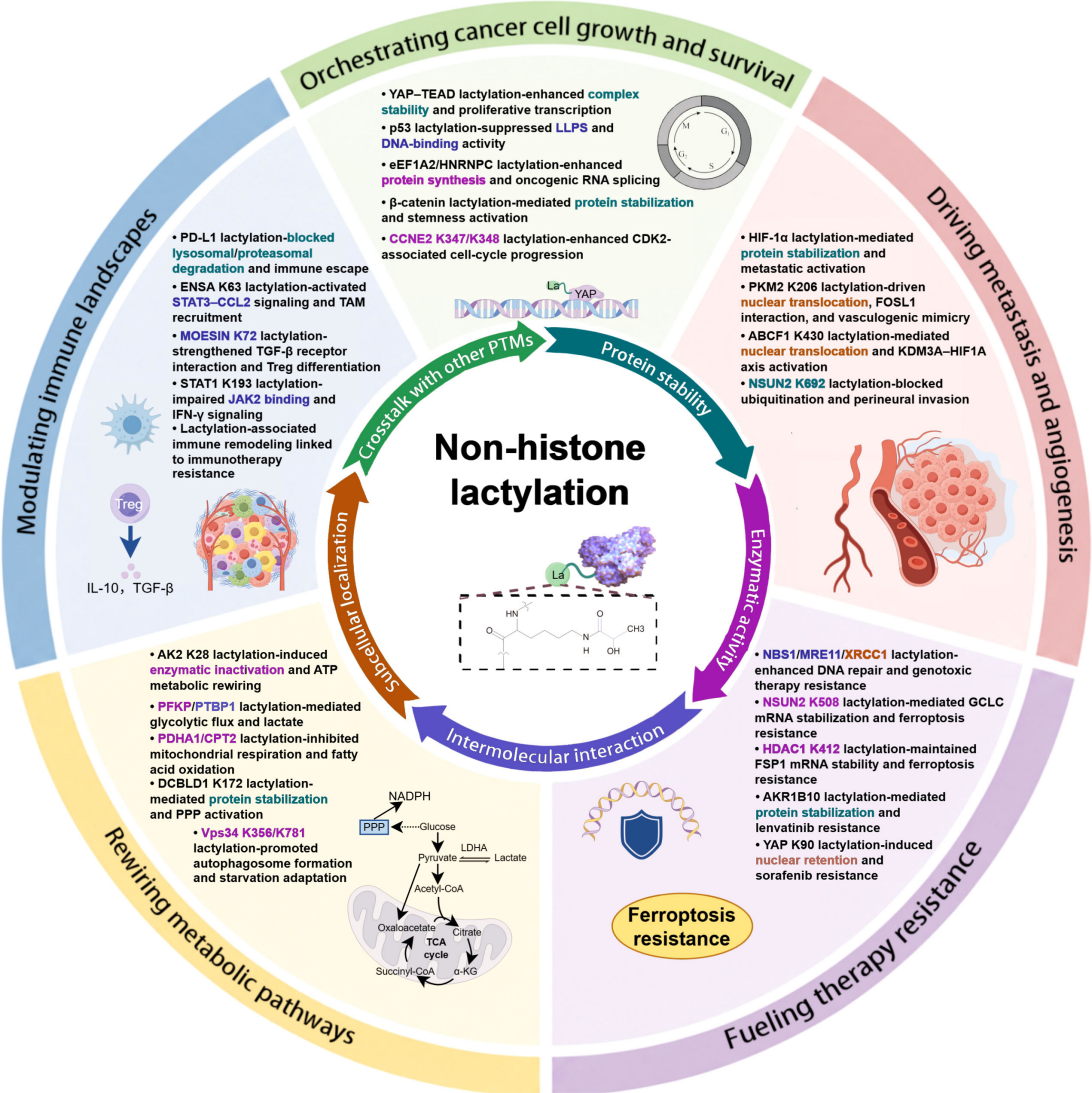
Multi-tier circular diagram of non−histone lactylation in cancer hallmarks. This diagram illustrates how non−histone lactylation (Kla) drives multiple cancer hallmarks. Center: Lactate−derived Kla. Middle layer: Five protein-level functional consequences, including altered protein stability, enzymatic activity, subcellular localization, interaction networks, and crosstalk with other PTMs. Outer layer: Five core cancer hallmarks, including proliferation, metastasis, therapy resistance, metabolic rewiring, and immune modulation. Specific non−histone substrates (e.g., CCNE2, PKM2, NBS1, PFKP, PD−L1) are placed within their respective hallmark sectors. A color-coding strategy visually links each protein to its corresponding functional consequence in the middle tier, emphasizing lactylation as an integrative hub connecting metabolism to signaling and therapeutic vulnerability.

The advent of sophisticated sequencing technologies, including lactylome and proteome analyses, has unveiled a rapidly expanding catalogue of lactylation-modified proteins. Accumulating evidence highlights the multifaceted roles of non-histone lactylation across multiple cancer types (46), acting as an integrative signaling hub that dynamically bridges molecular functional consequences with diverse cancer hallmarks (**Figure 3**). Notably, non-histone lactylation can also serve as an independent prognostic predictor (70). Additionally, studies have demonstrated that non-histone lactylation is involved in numerous intricate feedback regulatory mechanisms, further complicating the regulatory role of lactylation in cancer progression (47, 98). Understanding lactylation is thus crucial for advancing our knowledge of cancer and developing novel therapeutic strategies.

### Orchestrating cancer cell growth and survival

The Hippo pathway effectors YAP (Yes-associated protein) and TAZ (transcriptional co-activator with PDZ-binding motif), frequently dysregulated in various cancers, such as lung, colorectal, breast, and pancreatic cancers, are pivotal tumor-promoters responsible for cell growth and survival (112). Upon activation, YAP/TAZ translocate to the nucleus to form complexes with TEAD (TEA domain) transcription factors (113), promoting pro-survival and pro-proliferative gene expression, which contributes to tumor growth, invasion, metastasis, and resistance to treatments (114, 115). Recent work by Ju et al. identified AARS1/2 as lactate-sensing lactyltransferases that mediate overall lysine lactylation (46). AARS1 expression is elevated in gastric cancer (GC) specimens, and is associated with poor prognosis in GC patients. Intriguingly, AARS1-mediated lactylation stabilizes the YAP-TEAD complex, enhancing transcription of proliferation genes and the expression of Hippo target gene AARS1, constituting a feedforward loop to synergistically promote gastric cancer progression.

P53 is a key tumor suppressor that mediates multiple functions, such as DNA repair, cell cycle progression, and cell survival under stress, and its dysfunction is implicated in a significant proportion of human cancers (116). A recent study indicated that AARS1 can also catalyze the lactylation of p53 at lysines within its DNA-binding domain, which inhibits the liquid-liquid phase separation (LLPS) of p53, reduces its DNA-binding ability and alters downstream gene expression, thereby promoting the occurrence and development of cancer (45).

Parallel studies reveal the role of lactylation in cancer cell growth and survival through synergistic epigenetic and translational regulation mechanisms. METTL16, a well-characterized m6A methyltransferase, has been extensively investigated as a promising therapeutic target for cancer, with evidence suggesting its role in promoting cancer cell growth and accelerating cancer progression (117, 118). Recent study has shown that METTL16 plays a pivotal role in cuproptosis by targeting FDX1 (a copper reductase). Under copper stress, METTL16 undergoes K229 lactylation, which amplifies its enzymatic activity to enhance methylation of downstream genes, subsequently driving copper-dependent apoptosis (54). In colorectal cancer (CRC), lysine acetyltransferase 8 (KAT8)-mediated lactylation of translation elongation factor eEF1A2 at K408 (eEF1A2 K408la) accelerates protein synthesis and tumor cell proliferation based on *in vivo* and *in vitro* models (119). Additionally, lactylation also impacts RNA alternative splicing. In pancreatic cancer, heterogeneous nuclear ribonucleoprotein C (HNRNPC) is lactylated at lysine 176 (K176la). This modification strengthens HNRNPC binding to poly−U motifs in p21−activated kinase 6 (PAK6) pre−mRNA, upregulating the expression of the oncogenic isoform PAK6S and thereby promoting tumor growth (120).

Stemness and cell cycle control are pivotal features that drive cell proliferation and survival, and their dysregulation is often implicated in the malignant behaviors of cancer cells. In CRC cells, β-catenin, a key protein involved in the Wnt signaling pathway, is highly expressed and undergoes lactylation under hypoxic conditions, which enhances its stability and promotes cell proliferation and stemness (121). Similarly, lactylation also plays a significant role in subverting cell cycle control. Cyclin E2 (CCNE2), a key cell cycle protein that regulates the G1/S phase transition, mainly binds to cell cycle-dependent kinase 2 (CDK2), promoting uncontrolled cell cycle progression and DNA replication (122). In normal tissues, CCNE2 expression is tightly regulated, but it is often abnormally upregulated in various cancers, including hepatocellular carcinoma (HCC), breast cancer, and prostate cancer (123). Recent studies have shown that lactylation of CCNE2 at lysine residues K347 and K348 enhances its activity, thereby amplifying CDK2-driven proliferation in HCC (55). Interestingly, the enzyme SIRT3 has been identified as a potential counteracting factor in this process. SIRT3-mediated delactylation of CCNE2 counteracts the effects of lactylation, highlighting the therapeutic potential of SIRT3 activators in combating cancers. Collectively, these findings exemplify how lactylation intersects with malignant behaviors of cancer cells.

### Driving metastasis and angiogenesis

Lactylation emerges as a pivotal regulatory mechanism bridging tumor angiogenesis, invasion, and metastasis, with hypoxia-inducible factor 1α (HIF-1α) serving as a central molecular hub. Classically, HIF-1α is stabilized under hypoxia but is typically degraded via the ubiquitin-proteasome pathway under normoxia (124). In many solid tumors, such as lung cancer, liver cancer, and prostate cancer, HIF-1α is capable of escaping this degradation, driving angiogenesis and metabolic reprogramming to enhance metastasis. Recent studies reveal that lactylation subverts this canonical regulation. For instance, MCT1-mediated lactate influx induces HIF-1α lactylation under normoxic conditions, stabilizing it to activate metastatic programs (70). This lactylation-driven stabilization upregulates KIAA1199, a crucial pro-metastatic oncogene linked to poor prognosis (125), thereby enhancing angiogenesis and vasculogenic mimicry (VM) in prostate cancer (70). Beyond HIF-1α−regulated VM, lactylation directly drives VM through other mechanisms. In colorectal cancer, AARS1−mediated lactylation of PKM2 at K206 promotes PKM2 nuclear translocation and interaction with FOSL1, facilitating FOSL1−dependent super−enhancer formation and VM, while also contributing to bevacizumab resistance (126). Regarding HIF1A regulation, in hepatocellular carcinoma (HCC), lactylation of ABCF1 at lysine 430 (ABCF1-K430la) promotes its nuclear translocation via the KPNA2-KPNB1-RAN pathway, where it activates the KDM3A-H3K9me2-HIF1A axis to fuel lung metastasis (127).

Lactylation also operates through non-transcriptional mechanisms. In HCC, lactylation of adenylate kinase 2 (AK2) at K28 inhibits its function, further accelerating cancer progression (11). Moreover, a unique mode of metastasis is perineural invasion (PNI), a hallmark of pancreatic ductal adenocarcinoma (PDAC). Lactylation of NSUN2 at K692 stabilizes the protein by blocking its ubiquitination, enhancing m5C modification of CDCP1 and STC1 mRNAs, and promoting tumor-nerve interactions. This lactate-NSUN2-m5C-CDCP1/STC1 axis critically drives PNI and represents an actionable therapeutic target in PDAC (105).

### Fueling therapy resistance

The development of treatment resistance in tumor cells has long been a significant challenge in cancer therapy. Recently, emerging evidence highlights lactylation as a pivotal post-translational modification driving cancer therapeutic resistance through multifaceted mechanisms.

Lactylation of DNA repair machinery components serves as one of the major mechanisms underpinning resistance to genotoxic therapies. Chen et al. observed that lactic acid-pretreated cancer cells developed resistance to subsequent therapeutic interventions, with both ionizing radiation (IR) and cisplatin demonstrating reduced efficacy in the experimental model. Following their initial observations, the research team systematically investigated DNA damage repair pathways and evaluated the role of lactate in this process, uncovering that non-histone protein lactylation confers resistance across diverse therapies. To investigate the lactylation-dependent resistance mechanism, researchers performed proteomics analysis to explore lactylated substrates responsible for DNA repair, identifying NBS1 as the pivotal factor to promote homologous recombination (HR)-mediated DNA repair (56). Lactylation of NBS1 at lysine 388 (NBS1 K388) is crucial for the formation of MRE11-RAD50-NBS1 complex, enhancing DNA repair and thereby blunting the efficacy of cisplatin and radiation. The NBS1 K388R mutant abolishes lactylation at this critical site, thereby abrogating lactate-mediated potentiation of DNA repair in drug-resistant models, mechanistically linking K388 lactylation to enhanced MRE11-RAD50-NBS1 complex assembly and subsequent activation of DNA damage response pathways following double-strand break (DSB) induction (56). Further mechanistic dissection uncovered that CBP acetyltransferase orchestrates lactylation of MRE11 at K673 in response to DNA damage, specifically enhancing DNA binding affinity and resection efficiency. Remarkably, while MRE11 K673 lactylation promotes chemoresistance by accelerating HR repair completion after cisplatin and PARPi treatment, acetylation at this residue exhibited no measurable impact on resection dynamics, establishing lactylation as a distinct epigenetic regulator of DNA repair fidelity in chemoresistance (85). Additionally, XRCC1 lactylation at K247 also promotes DNA repair capacity, further contributing to chemoresistance in glioblastoma (91).

Beyond DNA repair machinery, lactylation also exerts ferroptosis-suppressive effects through metabolic-epigenetic crosstalk. Ferroptosis, a form of regulated cell death (RCD), is driven by iron-dependent lipid peroxidation (128), while resistance to ferroptosis is a typical phenotype exhibited by cancer cells during tumor progression (129, 130). In a recent study, Niu et al. elucidated the mechanism of the NAA10-NSUN2-GCLC signaling axis, which antagonizes doxorubicin-induced ferroptosis in the acidic state (131). Acidic microenvironments trigger NSUN2 activation via K508 lactylation, which stabilizes GCLC mRNA through m5C methylation to boost glutathione synthesis, effectively enhancing cellular antioxidant capacity and inhibiting lipid peroxidation, thereby reducing ferroptosis in drug-exposed tumors. Parallel investigation in colorectal cancer further reveals lactylation as a gatekeeper of ferroptosis resistance, which limits the efficacy of ferroptosis-based therapies. Yang et al. reveal the close relationship between histone deacetylase inhibitors (HDACi) and CRC resistance to ferroptosis (132). Both Vorinostat (SAHA) and Trichostatin A (TSA), HDACi specifically targeting HDAC1, notably reduce HDAC1 K412 lactylation, further restoring cell susceptibility to ferroptosis. Mechanistically, SAHA/TSA-inhibited HDAC1 K412 lactylation promotes the H3K27ac modification and activation of FTO (fat mass and obesity-associated gene) and ALKBH5 (RNA demethylase), which further destabilizes FSP1 mRNA via m6A demethylase activation. Therefore, combining HDACi with ferroptosis inducers holds promise as a therapeutic strategy for CRC.

This regulatory paradigm also extends to targeted therapy resistance. For example, YAP-K90 lactylation exemplifies a spatial regulatory mechanism, which creates a cryptic nuclear export signal (NES), forcing YAP nuclear retention through enhancing binding affinity with CRM1, subsequently amplifying HCC stemness and sorafenib resistance (133). In colorectal cancer, PKM2 K206la not only promotes vasculogenic mimicry but also confers resistance to the anti-VEGF antibody bevacizumab. Genetic or pharmacological inhibition of PKM2 lactylation disrupts VM and synergizes with bevacizumab in patient-derived preclinical models, significantly improving therapeutic efficacy (126). In hepatocellular carcinoma (HCC), the aldo-keto reductase AKR1B10 is stabilized by AARS1-mediated lactylation at lysine 173 (K173la), which blocks its ubiquitin-proteasomal degradation. Stabilized AKR1B10 interacts with LDHA and promotes LDHA Y10 phosphorylation, accelerating glycolytic lactate production. The increased lactate then induces histone H3K18 lactylation, which transcriptionally upregulates LDHA expression, forming a self-reinforcing positive feedback loop that drives lenvatinib resistance. Targeting this axis with the AKR1B10 inhibitor epalrestat synergizes with lenvatinib to overcome resistance in mouse xenograft and patient-derived xenograft models (134).

Collectively, these findings establish lactylation as a critical epigenetic driver of pan-therapeutic resistance, positioning anti-lactylation strategies as promising chemosensitization tools across various malignancies.

### Rewiring metabolic pathways

Lactylation has emerged as a central metabolic rheostat in cancer biology. This pervasive modification can directly target extensive metabolic enzymes involved in the tricarboxylic acid (TCA) cycle, fatty acid, amino acid, and nucleotide metabolism, primarily reprogramming cellular energy flux through three key mechanisms (11).

Glycolytic plasticity is dynamically tuned by lactylation. For instance, K28 lactylation inactivates AK2, fueling HCC proliferation and metastasis by redirecting ATP metabolism (11). Conversely, PFKP lactylation is regulated in a context-dependent manner. In fetal hepatocyte cells (FHC), PFKP lactylation inhibits its enzyme activity, which results in weakened glycolytic flux and reduced lactate production, consequently forming a lactate-driven negative feedback loop (135). However, lactylation of PFKP at K392 enhances glycolysis in ovarian cancer through PTEN suppression, further contributing to cancer progression (136). Another important regulatory enzyme in the glycolytic pathway, PFKFB4 (6-phosphofructose-2-kinase/fructose-2,6-bisphosphatase 4), can be regulated through AKT, mTOR, and HIF-1 signaling pathways (137–139). Recently, high levels of PTBP1-K436 lactylation were identified in glioma stem cells (GSCs), which stabilizes PFKFB4 mRNA to amplify glycolytic output and tumor aggressiveness (140).

Lactylation is also responsible for mitochondrial reprogramming. Hypoxia-induced PDHA1-K336 and CPT2-K457/8 lactylation inactivates their enzymatic activities, disrupting OXPHOS by suppressing acetyl-CoA efflux during pyruvate and fatty acid oxidation, whereas SIRT3-mediated delactylation reactivates mitochondrial respiration (47). This suppression is reversible through SIRT3-mediated delactylation, which reactivates mitochondrial respiration during nutrient repletion.

Lactylation-mediated anabolic support is crucial for fueling cancer progression through coordinated nutrient stress responses. For example, lactate enhances the expression of DCBLD1 through dual regulatory mechanisms (71). HIF-1α drives the transcriptional upregulation of DCBLD1, while DCBLD1 lactylation at K172 further promotes its protein stabilization. The overexpressed DCBLD1 physically shields glucose-6-phosphate dehydrogenase (G6PD) from autophagic degradation, thereby amplifying both G6PD protein abundance and enzymatic activity. The resultant pentose phosphate pathway (PPP) activation boosts NADPH/ribose-5-phosphate production, providing essential biosynthetic precursors for nucleotide synthesis and redox homeostasis. This lactylation-anchored metabolic network directly correlates with metastatic potency in cervical cancer models. Conversely, pharmacological PPP inhibition via 6-aminonicotinamide (6-An) suppresses G6PD activity, reversing lactylation-mediated anabolic advantages and restoring tumor susceptibility to nucleotide depletion therapies. Nutrient stress adaptation further integrates lactylation through ULK1-LDHA-driven lactate surges. Specifically, ULK1 orchestrates metabolic adaptation by phosphorylating LDHA at Ser196 under nutrient-deprived conditions, promoting lactate production. This lactate pool is co-opted by the acyltransferase KAT5/TIP60, which catalyzes dual lactylation of Vps34 at lysines 356 and 781, enhancing autophagosome formation and sustaining metabolic flexibility during starvation (98).

These findings collectively identify lactylation as a dynamic metabolic orchestrator, toggling between glycolysis, mitochondrial function, and anabolic pathways to maintain tumor bioenergetic homeostasis.

### Modulating immune landscapes

Beyond the well−known immunosuppressive effects of lactate accumulation in the TME, lactylation of non−histone proteins further positions lactate as a master modulator that reprograms immune cell functionality across malignancies.

In gastric cancer, integrative omics analyses using data from TCGA and GSE84437 cohorts identified six lactylation-associated hub genes, enabling the construction of a lactylation score model that stratifies patients into distinct prognostic groups. Significantly, high-lactylation tumors exhibit elevated genomic instability, tumor mutation load (TMB), and immunosuppressive infiltration, features that correlate with immunotherapy resistance, revealing the predictive role and therapeutic prospect of lactylation (141). Similarly, a recent study in breast cancer analyzed the expression pattern of 22 lactylation-related genes from TCGA and GSE20685 databases. Using principal component analysis (PCA), two lactylation clusters were identified, with different gene expression, survival outcomes, and immune microenvironments. A seven-gene lactylation prognostic model was constructed that predicts patient survival and stratifies therapeutic vulnerabilities: low-lactylation tumors may benefit from targeted therapies, while high-lactylation subtypes respond better to chemotherapy (142). These findings offer novel insights for precise management of breast cancer.

At the molecular interface, lactylation directly subverts immune surveillance by modulating checkpoint dynamics. In colorectal cancer cells, high lactate induces PD-L1 lactylation at lysine 810-813, which is catalyzed by GCN5 acetyltransferase and counteracted by SIRT5 deacetylase (143). PD-L1 lactylation elevates its protein stability by blocking lysosomal degradation, which amplifies PD-L1 membrane clustering, enables tumor cells to evade CD8+ T cell cytotoxicity, and induces immune escape. Moreover, PD−L1 also undergoes AARS1−mediated lactylation at K280, which inhibits HUWE1−dependent ubiquitination and proteasomal degradation, thereby stabilizing PD−L1 and promoting immune evasion. This modification correlates with poor prognosis in non−small cell lung cancer and represents a potential diagnostic biomarker (144). Additionally, PD-L1 lactylation enhances tumor cell tolerance to immune checkpoint inhibitors (ICIs), contributing to anti-PD-1 therapy resistance.

Beyond direct checkpoint modulation, lactylation can reprogram immune cell function. In pancreatic ductal adenocarcinoma (PDAC), clinical analyses reveal that elevated global protein lactylation, evidenced by ^18^F-FDG uptake, correlates with diminished immunotherapy efficacy and poor prognosis (86). Mechanistically, lactate-induced K63 lactylation of Endosulfine alpha (ENSA-K63la) in tumor cells activates STAT3-CCL2 signaling, recruiting TAMs to the TME. Concurrently, lactate also reprograms TAMs via ENSA-STAT3 to adopt an immunosuppressive phenotype, fostering resistance to immune checkpoint blockade (ICB). Targeting ENSA-K63la or CCL2 disrupts this axis, restoring TME immunogenicity and sensitizing PDAC to ICB in preclinical models (86). Additionally, lactylation modulates immune evasion by polarizing TAMs, creating an immunosuppressive niche permissive for metastatic spread (145). Another study focuses on the effect of lactylation on anti-PD-1 treatment through modulating Treg cell function. MOESIN lactylation at K72 strengthens its interaction with transforming growth factor β (TGF-β) receptor I, which hyperactivates SMAD3 to drive Treg differentiation, impairing antitumor immunity, and creating an immunosuppressive niche (82). Notably, the degree of MOESIN lactylation in Treg cells inversely correlates with the response to anti-PD-1 therapy, while combination of anti-PD-1 with lactate dehydrogenase inhibitors that block MOESIN lactylation synergistically enhances tumor control.

Interferon-γ (IFN-γ) signaling represents another key pathway suppressed by lactylation. AARS1 mediates lactylation of STAT1 at lysine 193 (K193la), which inhibits STAT1 binding to JAK2 and its phosphorylation, disrupting tumor responsiveness to IFN-γ and reducing downstream chemokines such as CXCL9, CXCL10, and CXCL11, ultimately facilitating immune escape (146).

Beyond oncology, lactylation can also compromise antiviral defenses. In HSV-1-infected models, cGAS lactylation disrupts STING signaling, subverting antiviral immune surveillance and permitting viral replication. However, blocking lactate metabolism via MCT1 inhibition can reverse this defect (44).

Collectively, these findings underscore that non-histone lactylation operates as a linchpin of tumor-immune metabolic symbiosis, driving immune evasion primarily through checkpoint stabilization, suppressive cell recruitment, and effector T cell dysfunction. Therapeutic strategies targeting lactylation regulators or downstream effectors may dismantle these immunosuppressive circuits, enhancing immunotherapy efficacy across diverse malignancies.

## Clinical implications

The expanding understanding of non-histone lactylation has unveiled novel therapeutic vulnerabilities across cancer types, driven by insights from multi-omics analyses and functional studies. Beyond prognostication, pharmacological modulation of lactylation networks via glycolytic enzyme inhibitors, lactylation-modifying agents, and drug repurposing offers actionable strategies to reverse therapy resistance.

### Lactate concentration as a prognostic biomarker

Beyond its role as a lactylation substrate, the concentration of lactate itself has long been recognized as a clinically relevant biomarker. In cervical cancer, primary tumors with lactate concentrations exceeding 20 μmol/g exhibit significantly elevated metastatic risk, and patients with higher lactate levels have diminished survival odds (147, 148), solidifying lactate as a valuable predictive marker for metastatic potential and survival. Similarly, in stage IV non−small cell lung cancer, elevated serum lactate dehydrogenase (LDH), a proxy for lactate production, strongly correlates with systemic tumor spread and reduced overall survival (149). These findings position lactate as a valuable prognostic marker, which may complement more specific lactylation−based signatures in clinical practice.

### Lactylation as a predictive biomarker for ICI response and prognosis

Lactylation has emerged as a critical oncogenic PTM across diverse malignancies, such as gastric cancer, lung cancer, and colorectal cancer, where heightened lactylation correlates with higher metastatic propensity and poorer survival (98, 150, 151). For instance, colorectal cancer patients with high lactate levels experience higher recurrence rates, distant metastasis, and advanced-stage progression (151), while HCC cohorts from Tongji Hospital reveal that elevated IGF2BP3 lactylation levels predict poorer progression-free and overall survival (152). Prognostic models leveraging lactylation-related genes (LRGs) are further reshaping cancer stratification. Yang et al. established a six-gene lactate score in gastric cancer linking high lactylation scores to malignant progression and poor prognosis (141), while a 16 LRG-based prediction model in multiple myeloma can effectively stratify patients by chemotherapy resistance, immune infiltration, and mortality risks (153). Similarly, lactylation-driven risk scores in HCC, ovarian cancer, and pancreatic ductal adenocarcinoma highlight its pan-cancer utility in predicting therapy resistance and tumor evolution (154–156).

Crucially, emerging evidence has linked lactylation status to ICI efficacy. In colorectal cancer (CRC), Huang et al. integrated IHC data from tissue microarrays and immunofluorescence analysis on colorectal cell lines, revealing the prevalent elevation of global lactylation. Furthermore, a 23-gene lactylation-related risk model was established, which not only distinguishes prognostic subgroups but also predicts differential immunotherapy responses and drug sensitivity (151).

These findings position lactylation signature as a prognostic predictor, as well as a biomarker for patient stratification and therapeutic optimization. However, challenges persist: pan-cancer analyses across 33 tumor types reveal context-dependent prognostic correlations, underscoring the need for tumor-specific validation (157). Additionally, the absence of population-level assays and limited evidence for lactylation as an early diagnostic biomarker necessitate large-scale clinical studies to further validate its utility in patient stratification and therapeutic targeting.

### Targeting glycolysis and lactylation processes

Altered lactylation status creates metabolic dependencies, making the inhibition of glycolysis-driven lactate production a cornerstone for lactylation suppression. Targeting key glycolytic enzymes can disrupt the Warburg effect to reduce lactate production, which might indirectly dampen lactylation and potentially reverse the immunosuppressive TME to hinder cancer progression.

2-deoxy-D-glucose (2-DG), a synthetic glucose analog to inhibit glycolysis, suppresses lactate production by disrupting glucose metabolism (158), effectively reducing lactylation in endometrial cancer, lung cancer, and hepatocellular carcinoma (159–161). However, the clinical translation of 2-DG is bottlenecked by its short half-life and dose-limiting systemic toxicities, such as hypoglycemia, fatigue, and gastrointestinal adverse effects, necessitating the development of optimized derivatives (162). LDHA is a crucial enzyme responsible for converting pyruvate to lactate. Inhibition of LDHA via inhibitor FX11 or genetic knockdown effectively reduces intracellular lactate levels, which stabilizes cGAS by attenuating its lactylation-dependent proteasomal degradation (73), further restoring the function of cGAS in STING signaling activation and IFN production (163, 164). Therefore, LDHA inhibition can amplify IFN responses and suppress tumor progression, linking metabolic elements with regulation of DNA-triggered innate immune response. However, the clinical advancement of many small-molecule LDHA inhibitors is severely hindered by distinct biochemical and pharmacokinetic hurdles. For instance, while FX11 exhibits therapeutic potential, its highly reactive catechol structure triggers non-specific, off-target effects that render it unsuitable for further clinical development (165). Furthermore, other promising classes of LDHA inhibitors, such as quinoline 3-sulfonamides, are plagued by critical *in vivo* pharmacokinetic defects, including poor body clearance and incompatible oral bioavailability (166).

Beyond glycolysis, targeting lactate transport mechanisms offers another promising strategy. Monocarboxylate transporter (MCTs) inhibitors can inhibit lactate efflux from tumor cells to reduce the extracellular lactate concentration and lactylation in immune cells, thereby alleviating the immunosuppressive effects of lactate in the TME (167). For example, the MCT1 inhibitor AZD3965 has completed phase I clinical trials for the treatment of advanced solid tumors and lymphomas, showing tolerability at sufficient doses to achieve target binding (168). This not only starves neighboring immune cells of immunosuppressive lactate but also reduces lactylation modifications in TAMs and cytotoxic T cells, revitalizing antitumor immunity. Nevertheless, the trial also identified dose-limiting toxicities, such as retinopathy, cardiac troponin elevation, and acidosis, consistent with on-target MCT1 expression in the retina and heart, necessitating careful dose titration and ophthalmologic monitoring in future studies (168).

Direct lactylation modulation is also achievable through inhibitors targeting lactylation “writers” (e.g., CBP/p300) and “erasers” (e.g., HDAC). For instance, CBP inhibition blocks MRE11 lactylation, which impedes HR and sensitizes tumors to cisplatin and PARP inhibitors (olaparib) (85). The HDAC inhibitor SAHA and TSA notably suppress HDAC1 lactylation (K412), restoring cell sensitivity to ferroptosis inducers (e.g., RSL3, erastin) in CRC models (132). Significantly, the combination of HDACi and ferroptosis inducers achieves efficacy comparable to standard chemotherapy (5-fluorouracil + oxaliplatin), with better safety and no significant adverse effects, revealing a promising novel strategy for CRC treatment. Nevertheless, the broad-spectrum nature of pan-HDAC inhibitors frequently results in a lack of substrate specificity and dose-limiting hematological toxicities, particularly thrombocytopenia and neutropenia, which narrow their therapeutic windows (169, 170).

A direct comparison reveals distinct toxicity profiles: 2-DG causes hypoglycemia; FX11 suffers from off-target catechol reactivity; AZD3965 leads to retinopathy and cardiac effects; and pan-HDAC inhibitors induce thrombocytopenia and neutropenia. While targeting the lactate-lactylation axis presents a promising therapeutic frontier, its clinical translation is currently bottlenecked by a convergence of pharmacological and biological bottlenecks. The upstream metabolic inhibitors induce dose-limiting systemic toxicities due to the ubiquitous reliance of normal tissues on glycolysis, while downstream transport inhibitors exhibit tissue-specific accumulation and narrow therapeutic windows. Furthermore, the advancement of targeted agents is frequently derailed by *in vivo* pharmacokinetic defects and chemical hyper-reactivity, as well as a lack of enzymatic substrate specificity leading to hematological toxicity. The overarching challenge lies in the inability of current systemic therapies to distinguish between the metabolic dependencies of tumor cells and highly active normal tissues. Therefore, overcoming these dose-limiting factors will require a multi-faceted approach, such as structural optimization of small molecules to enhance bioavailability, the development of sophisticated tumor-targeted delivery systems, and the implementation of rational combination therapies to achieve maximum anti-lactylation efficacy at lower, tolerable doses.

### Targeting lactylation effectors

Cell-penetrating peptides (CPPs) represent a cutting-edge and effective strategy to specifically disrupt post-translational modification of target proteins. For example, CPPs designed to block MRE11 lactylation have been shown to synergize with anti-tumor therapies, enhancing cancer cell sensitivity to olaparib and cisplatin (85). Peptides targeting ENSA-K63la reduce ENSA lactylation and disrupts STAT3-CCL2 signaling in PDAC, reprogramming the TME and sensitizing anti-PD1 immunotherapy (86). Similarly, a peptide targeting STAT1 K193la (K193-pe) restores IFN-γ signaling and enhances CD8^+^ T cell recruitment to improve immune checkpoint blockade efficacy (146). Beyond immunomodulation, a peptide inhibiting ERK K231la (ERK-lac peptide) impairs tumor growth in KRAS-mutant cancer models (171), and a peptide targeting NOL6 K54 lactylation (K54-pe4) suppresses CRC proliferation and metastasis (106). Collectively, these examples underscore the feasibility and therapeutic potential of substrate-specific lactylation inhibition without broad metabolic disruption.

### Drug repurposing and combination therapies

Based on molecular docking and conformational analyses, several existing drugs with latent anti-lactylation activity have been identified, highlighting the potential for drug repurposing in cancer therapy (172). For example, Fargesin, a natural lignan, has been demonstrated to serve as a glycolysis inhibitor and suppress lactate production by targeting rate-limiting enzymes like PKM2, thereby effectively reducing lactylation in non-small cell lung cancer (173). Additionally, SIRT3, identified as a delactylase, can be activated by the phytochemical Honokiol, which enhances apoptosis in HCC by modulating CCNE2 lactylation (55). Other examples include the antifungal agent itraconazole, which exhibits off-target inhibition of LDH (174), and the antidepressant fluoxetine, which modulates MCT4 expression (175). Therefore, repurposing such compounds, either as monotherapies or in combination with ICIs/chemotherapies, potentially offers a cost-effective pathway to accelerate clinical translation.

Moreover, the combination of glycolysis inhibitors with immunotherapy has also shown significant promise in enhancing anti-tumor responses across various cancer types (176). In NSCLC, combining oxamate (a LDHA inhibitor) with anti-PD-1 therapy can effectively inhibit proliferation of Ki67+ tumor cells by reducing intratumoral lactate levels (177). Similarly, in glioblastoma, the combination of oxamate with CAR-T cells extends survival in orthotopic tumor-bearing mice compared to CAR-T monotherapy (178). In HCC models, a typically immunoresistant malignancy (179), pharmacological reduction of lactate synergizes with anti-PD-1 treatment to enhance anti-tumor efficacy (82). Collectively, these findings underscore the broad applicability and potential benefits of metabolic-immune cotargeting strategies in diverse cancer types. Future research should focus on further exploring these combinations to optimize therapeutic outcomes and improve patient prognosis.

## Conclusion

Non-histone protein lactylation has emerged as a pivotal regulatory mechanism governing tumor cell proliferation, metastasis, and immune evasion. Mechanistically, lactylation orchestrates multifaceted functional reprogramming by modulating protein stability, disrupting interaction hubs, and rewiring subcellular trafficking. Crucially, lactylation intersects with other PTMs, creating complex regulatory networks within cancer cells. Therapeutically, universal lactylation elevation across malignancies correlates with immunosuppression and therapy resistance, while its regulatory machinery offers druggable targets. However, research in this field is still at an early stage, and key knowledge gaps persist, including the incomplete cataloging of lactylated proteomes, undefined substrate specificity of lactylation-modifying enzymes, and limited clinical translation of lactylation-targeted interventions. Further research is required to bridge these gaps and unlock paradigm-shifting therapies that simultaneously disrupt tumor-intrinsic adaptation and resurrect antitumor immunity, ultimately redefining precision oncology in the era of cancer metabolism.

## Glossary

TME: Tumor microenvironment
Kla: Lysine lactylation
PKM2: Pyruvate kinase M2
ATP: Adenosine triphosphate
TCA: Tricarboxylic acid
LDH: Lactate dehydrogenase
MCT: monocarboxylic acid transporter
CAF: cancer-associated fibroblasts
MSC: mesenchymal stem cell
CTL: Cytotoxic T cell
Treg: regulatory T cell
NET: neutrophil extracellular traps
ECM: Extracellular matrix
EMT: Epithelial-mesenchymal transition
OXPHOS: Oxidative phosphorylation
MDSC: Myeloid-derived suppressor cells
APC: Antigen-presenting cell
DC: Dendritic cells
GPR81: G protein-coupled receptor 81
HCAR1: Hydroxycarboxylate receptor 1
IFN: Type I interferon
HDAC: Histone deacetylase
cAMP: cyclic AMP
ARG1: arginase 1
VEGF: Vascular endothelial growth factor
MGO: Methylglyoxal
BRD: Bromodomain
TAM: Tumor-associated macrophage
LLPS: liquid-liquid phase separation
NSCLC: non-small cell lung cancer
m6A: N6-methyladenosine
PEP: Phosphoenolpyruvate
FASN: Fatty acid synthase
NAFLD: non-alcoholic fatty liver disease
MOESIN: Membrane-organizing extension spike protein
HR: homologous recombination
PDAC: Pancreatic ductal adenocarcinoma
ENSA: Endosulfine alpha
SAM: S-adenosylmethionine
GC: Gastric cancer
CRC: colorectal cancer
HCC: Hepatocellular carcinoma
IR: Ionizing radiation
RCD: Regulated cell death
TSA: Trichostatin A
NES: Nuclear export signal
PPP: Pentose phosphate pathway
PCA: Principal component analysis
CRC: Colorectal cancer
CPP: Cell-penetrating peptide
